# WRNexo is not required to maintain normal sex ratios in Drosophila: A CURE-Based Investigation

**DOI:** 10.17912/micropub.biology.001620

**Published:** 2025-06-09

**Authors:** Elyse Bolterstein, Shubhangee Mungre, Kara Nuss, Eric P. Stoffregen

**Affiliations:** 1 Biology, Northeastern Illinois University, Chicago, Illinois, United States; 2 Physical, Life, Movement & Sport Sciences Division, Lewis-Clark State College, Lewiston, Idaho, United States

## Abstract

*WRNexo* and *Blm* , Drosophila orthologs of human WRN and BLM RecQ helicases, play crucial roles in DNA replication and repair. Using a Course-based Undergraduate Research Experience (CURE) in an introductory Biology course, we investigated whether *WRNexo ^Δ^* mutants exhibit a progeny sex-bias similar to *Blm* mutants. Chi-square analyses revealed no deviation from expected Mendelian ratios or sex-bias among *WRNexo ^Δ^* offspring. These findings suggest that WRNexo does not affect sex-specific survival and may not be essential for replication of highly repetitive DNA sequences. Our study demonstrates that CUREs effectively engage students in hypothesis-driven research while contributing meaningfully to genomic stability studies.

**Figure 1. The WRNexo mutation does not contribute to differences in offspring Mendelian- or sex-ratios.**
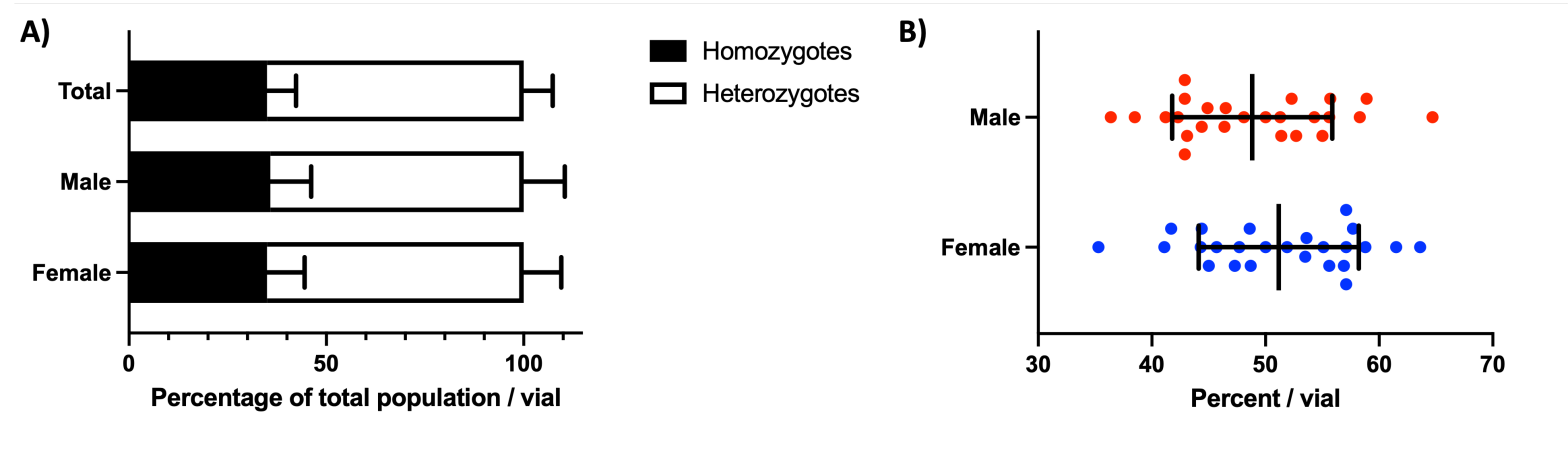
A) Progeny from *WRNexo ^Δ^* mutants emerge in Mendelian ratios (Total *Χ ^2^* = 0.745, *p* = 0.39; Male: *Χ ^2^* = 0.963, *p* = 0.326, Female: *Χ ^2^* = 0.278. *p* = 0.598; one-way ANOVA *p =* 0.965; n = 14 vials, mean 62 flies/vial). B) Progeny from *WRNexo ^Δ^* mutants emerge in predicted sex ratios ( *Χ ^2^* test: *Χ ^2^* = 1.808, *p* = 0.179; t-test: *p =* 0.246; n = 25 vials, mean 51 flies/vial)

## Description

BLM and WRN, members of the conserved RecQ helicase family, are critical in maintaining genomic stability through their roles in DNA repair, replication, transcription, and telomere maintenance. In addition to its RecQ helicase domain, WRN also contains an exonuclease domain with proofreading capabilities (Lu & Davis, 2021). WRN and BLM have been shown to coordinate with each other and other proteins during DNA repair and replication (Machwe et al., 2006, 2011; Pinto et al., 2016; Wu et al., 2018). Mutations in BLM and WRN lead to the autosomal recessive diseases Bloom Syndrome and Werner Syndrome, respectively. Patients with either syndrome have a predisposition to cancer; however, Werner Syndrome patients also show progeroid symptoms (Tsuge & Shimamoto, 2022). Additionally, WRN has been implicated in synthetic lethality in cancers with microsatellite instability (Chan et al., 2019; van Wietmarschen et al., 2020), further demonstrating the importance of these genes in preventing cancer and other diseases.

*Drosophila melanogaster* has highly conserved orthologs of both BLM and WRN, *Blm* and *WRNexo,* making it an excellent model for studying their DNA repair and replication functions. Notably, *WRNexo* contains only the exonuclease domain and not the helicase domain (Saunders, 2008; Bolterstein, 2014). Both Blm and WRNexo respond to replication stress during early development, as demonstrated by DNA damage during embryonic syncytial division, low hatching frequency, and sensitivity to hydroxyurea (McVey et al., 2007; Bolterstein et al., 2014), and both *Blm* and *WRNexo* are synthetically lethal with structure selective endonucleases (Andersen et al., 2011; Bolterstein et al., 2014). Together these data suggest coordination of these two proteins in flies, with more pronounced phenotypes in *Blm* mutants. Recently, *Blm* mutants were found to cause a maternal loading-dependent sex-bias during early embryogenesis, favoring female survival due to the lower repetitive DNA content in the XX karyotype compared to XY (Ruchert et al., 2022). This indicates Blm helicase is critical during rapid syncytial DNA replication. Given the proposed coordination between WRNexo and Blm, we hypothesized *WRNexo* mutants would also show a progeny sex-bias, though less pronounced than *Blm* mutants.

To investigate a progeny sex-bias in *WRNexo* mutants, we constructed a Course-based Undergraduate Research Experience (CURE) for the 84 students enrolled in General Biology I courses during a single semester at Northeastern Illinois University. CUREs effectively engage students in hypothesis-driven research and have been well documented to increase students’ skills in laboratory work, critical thinking, data analysis, and communication (Auchincloss et al., 2014; Brownell et al., 2015; Rodenbusch et al., 2016). Additionally, CUREs offer authentic research experiences to students unable to join traditional research programs. We designed a 3-week CURE that allowed students to practice the core General Biology concepts of Mendelian genetics and DNA replication using the following schedule: Week 1: Assess Mendelian ratios in *WRNexo* offspring; Week 2: Test for progeny sex-bias in *WRNexo* offspring; and Week 3: Practice PCR/electrophoresis by confirming the *WRNexo* mutation (addresses a general course learning outcome).

The students first confirmed that *WRNexo ^Δ^* deletion flies emerged in Mendelian ratios by counting progeny from crosses between two *WRNexo ^Δ^* heterozygotes. Students could easily determine offspring genotype based on eye color: heterozygotes displayed red eyes whereas homozygotes exhibited white eyes. The offspring from these crosses were composed of 35% homozygotes, which was not statistically different from the 33% expected for a cross with a lethal allele ( Figure 1A ; *Χ ^2^* = 0.745, *p* = 0.39). We further analyzed our data by sex and again found no statistically significant difference from expected ratios ( Figure 1A ). To investigate progeny sex-bias, we crossed homozygous *WRNexo ^Δ^* males and females and counted their offspring. While *WRNexo ^Δ^* homozygotes do exhibit a lower fecundity, they produce viable offspring (Bolterstein et al., 2014). Both male and female offspring were not statistically different from the expected 50% for each sex ( Figure 1B ). The above findings show that lack of *WRNexo* does not impact expected progeny sex ratios.

The lack of a progeny sex-bias in the offspring of *WRNexo ^Δ^* mothers suggests that, unlike Blm, *WRNexo* does not differentially impact genomes with additional repetitive DNA during syncytial S-phases. Unlike *Drosophila Blm, WRNexo* does not contain a RecQ helicase domain, limiting its capacity for unwinding secondary structures formed by highly repetitive DNA regions or restarting replication forks impeded in these regions. Therefore, it is unlikely that WRNexo plays a strong role in coordinating with Blm in resolving replication challenges caused by repetitive DNA sequences. The essential role of Blm during embryogenesis is limited to the maternally loaded protein needed for syncytial nuclear divisions (Ruchert et al., 2022). Because we did not find a progeny sex-bias in the complete absence of *WRNexo,* we did not further test whether maternal loading was important for this result.

This CURE engaged early-stage biology students while addressing a meaningful research question. Despite yielding a negative result, the project helped students connect key concepts like Mendelian genetics and DNA replication to real-world research. Students learned that unsupported hypotheses are a valuable part of science, fostering a growth mindset that supports future experimentation. Many students reported that this was one of their favorite labs in the class, citing enthusiasm for authentic research. Some even pursued additional research opportunities as a result. Beyond its educational value, the study offered new insight into the distinct roles of Blm and WRNexo in DNA replication, with broader implications for understanding cancer development, disease mechanisms, and potential therapeutic strategies.

## Methods

Fly husbandry and genetic crosses: All fly stocks were reared on solid cornmeal agar medium (BF Formula, Genesee Scientific) and maintained at 25°C under a 12-hour light/dark cycle. The *WRNexo ^Δ^* null mutants were generated previously in the lab (Bolterstein, 2014) and placed over a balancer that contained a *w ^+^* allele so that students could easily identify mutants by eye color ( *w;* *WRNexo ^Δ^* / *TM3 Ser {Act-GPF, w+}* ). For genetic crosses, virgin females were mated in narrow mouth vials with corresponding males; parents were removed after 3-4 days. Flies were counted two weeks after set-up to ensure all offspring had emerged.

Statistical analysis: Descriptive statistics and standard deviation for fly counts were calculated using GraphPad Prism, v10. Microsoft Excel was used to calculate Х ^2^ values.

## Reagents

**Table:** 

Strain	Stock	Source
* WRNexo ^Δ^*	*w;* *WRNexo ^Δ^* / *TM3 Ser {Act-GPF, w+}*	Bolterstein Lab (Bolterstein, 2014)
